# Metagenome of *Acropora palmata* coral rubble: Potential metabolic pathways and diversity in the reef ecosystem

**DOI:** 10.1371/journal.pone.0220117

**Published:** 2019-08-08

**Authors:** Andrés Sánchez-Quinto, Luisa I. Falcón

**Affiliations:** 1 Posgrado en Ciencias del Mar y Limnología, Universidad Nacional Autónoma de México, UNAM, Ciudad de México, México; 2 Laboratorio de Ecología Bacteriana, Instituto de Ecología, UNAM, CDMX, México; University of Bologna, ITALY

## Abstract

Over the past 30 years, the stony coral *Acropora palmata* has experienced an excessive loss of individuals showing few signs of recovery throughout the Mexican Caribbean, resulting in long stretches of coral rubble structures. When the coral dies, the skeleton begins to be colonized by algae, sponges, virus, bacteria and other microorganisms, forming a new community. Here we analyze, using a metagenomic approach, the diversity and biogeochemical cycles associated to coral rubble in La Bocana (Puerto Morelos, QRoo, Mexico). This study provides the first broad characterization of coral rubble associated communities and their role in biogeochemical cycling, suggesting a potential view of a world where coral reefs are no longer dominated by corals.

## Introduction

Tropical coral reef ecosystems are often referred to as the rainforests of the oceans since they comprise only a small fraction of the bottom surface area, yet are estimated to provide habitat for over 25% of all marine species [1]. They are complex ecosystems consisting of a vast array of animals, plants, microorganisms, and viruses [2]. Coral reefs are formed by calcium carbonate skeletons secreted by stony corals. Other organisms such as algae and sponges, may play critical roles in the construction of these ecosystems [3]. They are extremely important for nutrient cycling in shallow, oligotrophic, tropical waters [4]. Hence, coral reefs are amongst the most biologically diverse and economically important ecosystems on the planet. They can provide ecosystem services including fisheries, coastal protection, building materials, new biochemical compounds, tourism, habitat and shelter for many organisms [5]. Reef productivity is largely dependent on the capture and recycling of nutrients and trace elements by reef-associated bacterial communities [4]. Moreover, healthy reefs are important for carbon and nitrogen fixation, providing sources of essential nutrients for the marine food chain [4]. Coral reef bacterial communities occupy a range of different habitats including the sediment, overlying water column, and benthic invertebrates such as corals and sponges [4].

Unfortunately, the impact of overfishing, coral bleaching and diseases, ocean acidification and other environmental change combinations are affecting the fitness of corals [5]. There are different perspectives on how degradation and loss of biological diversity affect the functions of coral reef ecosystems and their generation of system services. However, the ecological services of reef ecosystems are poorly understood and information related to these are scarce [6], especially in the Mexican Caribbean.

Over the past 30 years one coral genus (*Acropora* spp.) in particular has experienced dramatic declines in abundance, with few areas showing signs of recovery to date [7]. This decline in several areas of the Caribbean has been related to bleaching events, storms, neglectful tourism and diseases affecting the productivity, nutrient cycles, and health of the reefs [8–12]. Acroporids decline has great consequences in the functioning and structure of the reefs of the Mexican Caribbean since *Acropora palmata* combines branching morphology with high rates of calcification [13]. Currently, several dead *Acropora* rubble patches are abundant in the Mexican Caribbean [14].

Coral rubble is often composed of material derived from the dead branches that originate from the reef front. When coral breaks it can accumulate *in situ* and be transported by currents to form rubble ridges in the reef lagoon, resulting in a permanent cycle of coral destruction and regeneration [15]. Lithification by either biological or physical cement stabilizes the secondary reef structure and is involved in the composition and preservation of the rubble [16]. The role of coral rubble in reef development is not only the contribution of a significant amount of carbonate to the primary reef structure but also aiding in stabilization (binding) of the reef framework. This process begins to create a new reef structure that increases in extension according to the deterioration of healthy *Acropora palmata* corals. Beltrán et al [17] suggested that microbial calcification in *Acropora* rubble can be induced in biofilms composed of extracellular polymeric substances (e.g. polysaccharides, proteins, lipids, nucleic acids) and a variety of microorganisms attached to coral rubble, becoming an important cementing agent. They also reported microbial diversity differences between the coral rubble biofilms and adjacent biotypes such as the water column, a microbial mat, the sediment and healthy *A*. *palmata* [17].

The colonization of coral rubble biofilms may have enormous relevance in the ecological processes within the ecosystem and biogeochemical cycles, although there is not enough knowledge regarding the composition or the function of the coral rubble biofilms [18]. The importance of genomic research of coral rubble may help understand this new feature that is becoming more abundant in coral reef ecosystems. This study presents the first metagenomic survey of *A*. *palmata* rubble in La Bocana (Puerto Morelos, Mexico) aiming to determine its composition and potential ecological role in an emerging coral reef ecosystem.

## Materials and methods

### Study area

The Puerto Morelos reef is part of the reef barrier called "Mesoamerican Reef System", which is the second largest in the world [19] located north of the state of Quintana Roo, approximately 33 km south of Cancun and 35 km north of Playa del Carmen. The climate is warm sub-humid with an average annual temperature of 26.3°C, a maximum in the summer of 35.5°C and a minimum in winter of 13°C. It is characterized by a reef lagoon located at 20°52' 32” N and 86° 51' 37.79” W [19, 20]. The lagoon is defined by a coastline and by a coastal reef barrier of around 5.5 km in length; the distance varies between 350 and 1600 m from the coastline [21]. Lagoon depth ranges from 2–8 m with an average of 3.5 m [20]. The water inside the lagoon maintains a temperature between 31 ºC and 32 ºC in summer (August-September) and in winter it drops to 24 ºC - 25ºC (December-January). The average salinity is 35.7 and the water remains approximately at a pH of 8.19 [20].

**Fig 1:**
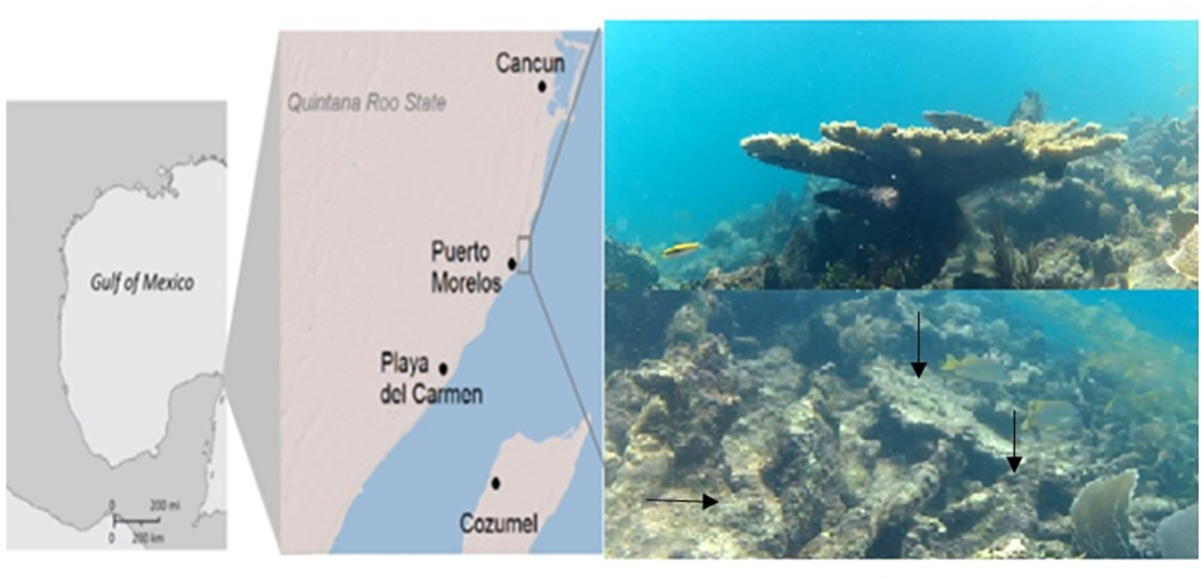
**Geographic location (left) of sampling points within the study area "La Bocana" in the reef system of Puerto Morelos, Quintana Roo (Upper-right inset shows a healthy *A. palmata* and in the lower-right *A. palmata* coral rubble structures).** Samples were collected using hammer and chisel.

This study focuses on the coral rubble of *A*. *palmata* which is a common feature in the reef formation called "La Bocana Grande" [17], located ~3 km north of the Academic Reefs Systems Unit (RSU) UNAM (Puerto Morelos, QRoo) (Fig 1). This reef has been identified as a region of high mortality of *A*. *palmata* and has the lowest living coral coverage in the National Park of Puerto Morelos [8, 22].

### Sample collection

All samples were obtained between July and August of 2015 from 9:00 h to 13:00 h using the Academic Reefs Systems Unit (RSU) boat. All samples were collected every 10 m in a single line transect to the north, from the starting point of 20°52' 32” N and 86° 51' 02” W with scuba diving equipment using a hammer, chisel and gloves. Samples of 6 different points were 3 replicates from the coral rubble of the "Bocana Grande" were collected from a 3–7 m depth (Fig 1). Part of the samples were kept on ice during the trip and at -20°C after arrival to the Reef Systems Unit, UNAM (RSU), while the other part were kept in water from the “Bocana Grande” for nitrogen fixation and methanogenesis assays.

### Nutrients

Six water samples for dissolved nutrient determinations were taken with sterile syringes. Samples were divided into two groups. For the first set of samples, 30 ml of pre-filtered water with nitrocellulose membranes (0.22 μm) were used, to which three drops of chloroform were added and kept frozen until analysis. The determination of the concentration of soluble reactive phosphorus (SRP), Ammonium, Nitrate and Nitrite was determined for this first set of samples, using a continuous flow analyzer (Skalar San Plus, Skalar) with the standard methods adapted by Grashoff et al [23] following the circuits of Kirkwood [24]. The second set of samples (not filtered water) were used for total nitrogen and total phosphorus analysis according to the Valderrama method [25]. The analyses were carried out with the support of the Laboratory of Aquatic Biogeochemistry, at the Institute of Marine Sciences and Limnology, UNAM.

### Elemental analysis (C, N and P) of coral rubble

For the elemental analysis of the coral rubble, superficial sections of approximately 1 cm^3^ were extracted and kept frozen and in the dark until their analysis. Subsequently, the samples were dried at 12°C with vacuum in the Savant SpeedVac drier (Whaltham, MA, USA) for at least 3 hours. Once dry, they were macerated with mortar and pestle to a fine powder. We obtained 20 mg of this mixture (n = 3 for each coral rubble sub-sample) to estimate the elemental composition of C and N using a PerkinElmer 2400 elemental Analyzer. For phosphorus estimation, an oxidation with persulfate at high temperature using the Valderrama [25] method was performed. To estimate the elemental composition of the organic component of the coral rubble, the carbonates were removed, incubating approximately 150 mg of the dry and macerated mixture of each rubble fragment with 1.5 N hydrochloric acid in test tubes. These were placed in grills at 40ºC inside the extraction hood. Once all the liquid was evaporated, the samples were resuspended in deionized water and centrifuged for 20 minutes at 4000 *g* at room temperature. The supernatant was recovered to analyze dissolved and particulate forms of N and P. The pellets were dried at 50°C and kept in a desiccator until analysis. The pellets of the organic component (~15 mg) were used for elemental analysis of C and N, as well as for the analysis by the Valderrama method of total nitrogen and total phosphorus. The analyses were carried out with the support of the Laboratory of Aquatic Biogeochemistry, at the Institute of Marine Sciences and Limnology, UNAM.

### Genomic extraction

Approximately 5 g of triplicate samples from six sites were extracted for total genomic content. Samples were macerated with liquid nitrogen and resuspended with extraction buffer (EDTA 0.25 M, NaCl 1.5 M, TRIS-HCl pH 8) and SDS (10%) repeating the freeze/thaw cycle three times, then incubated with lysozyme (30 mg/ml) for 30 minutes. Nucleic acids were extracted twice with phenol: chloroform: isoamyl alcohol (25: 24: 1) and once with chloroform: isoamyl alcohol (24: 1), recovering the supernatant after each centrifugation (13,000 rpm for 15 min). Subsequently, the aqueous phase of each sample was transferred to a sterile tube and a volume of isopropanol, 10% sodium acetate 3 M and 2 μl glycoblue was used to precipitate DNA o.n. at -20°C. Samples were centrifuged for 15 minutes at 13,000 rpm at room temperature. The supernatant was decanted and the pellet mixed with one mL of 70% ethanol, centrifuged at 13,000 rpm at room temperature for 5 minutes. The supernatant was decanted allowing the pellet to dry and resuspended in 30 μl of 1x TE buffer. All samples were run on 1% agarose gels to determine the quality of total DNA and all extractions were collected in a single 1.5 ml eppendorf tube reaching ~30 micrograms/mL. Concentration was measured using Qubit 2.0 Fluorometer.

### Metagenomic analysis

After the extraction, all samples were pooled to prepare a single metagenomic library with the Nextera DNA Flex library prep kit (Illumina, San Diego, CA) where fragments of total DNA (1 μg) were inserted into vectors and sequenced with whole genome sequencing technology (HiSeq 2 x 150), at the Yale Keck Center for Genome Analysis. We used two annotation strategies: MG-RAST [26] portal (https://www.mg-rast.org/index.html?stay=1) for taxonomical annotation and metabolic pathways and a “manual strategy” for identifying the organisms related to specific metabolic pathways. A total of 168,585,058 reads were recovered and 146,635,889 remained after quality filtering within MG-RAST. We removed all sequences from Enterobacteria phage phiX174 *sensu lato* since it is a common contamination in Illumina NGS [27]. Sequences obtained in this study are available in MG-RAST under the accession number (mgs696720).

For the “manual strategy” the quality of the sequences was evaluated with FASTQC [28]. Cuttings were made with Trimmomatic [29] to optimize the quality and they were reevaluated with FASTQC. Subsequently, the best assembly was compared using kmer from -22 to -99 with the programs: MEGAHIT [30], IDBA-UD [31] and metaSPAdes [32]. We decided to continue the next steps with the best quality assembly (IDBA-UD-1,952,389 contigs) evaluated by QUAST [33]. For gene prediction and read clustering, Prodigal (1,895,953 sequences) and CD-HIT (1,771,109 clusters) were used respectively [34, 35]. The annotation was performed using Ghost-Koala [36] (https://www.kegg.jp/ghostkoala/). To identify the organisms implicated in the pathways of interest in this study, which are those involved in biogeochemical cycling, we followed the annotation of “energy metabolism” classification within KEGG-pathways (https://www.genome.jp/kegg/pathway.html).

Moreover, a classification into pathways by unique genes of each biogeochemical pathway (nitrogen metabolism, sulfur metabolism, methane metabolism, carbon fixation and photosynthesis) was implemented (Table 1). Hence, genes found in another module from the same metabolic pathway were discarded. For example: for dissimilatory nitrate reduction. First, we searched for all genes per module using KO´s codes for NarGHI, NapAB, NirBD and NrfAH in the Ghost-Koala annotation to confirm that the route was complete. Then, using KEGG “nitrogen metabolism- Reference pathway” we selected only the specific genes (in this case only NirBD and NrfAH since NarGHI and NapAB are genes shared with the denitrification module). Finally, once having the specific genes per module, the abundance of each KO code were identified within the Ghost-Koala annotation to select the predominant organisms. For the dissimilatory nitrate reduction: Gammaproteobacteria, Planctomycetes and Nitrospirae were the abundant phyla, and *Planctomyces*, *Nitrospira* and *Cobetia* were the abundant genus.

**Table 1:** Metabolic pathways analyzed following the energy metabolism proposed by KEGG pathways.

Energy metabolism			
Methane metabolism	Searched genes		KO code
Methanotrophy	*pmoA-amoA-C*,*mmo-X*,*Y*,*Z*,*B*,*C*,*D*, *mdh1*,*mdh2*, *MOX*		K10944,K10945,K10946,K16157,K16158,K16159,K16160,K16161,K16162,K14028,K14029,K17066
Methanogenesis	*mcrA*, *B*, *G*, *C*, *D*		K00399, K00401, K00402, K03421, K03422
CO2→methane	*fwdA*,*B*,*C*,*D*,*E*,*F*,*G*,*H*, *ftr*, *mch*, *mtd*, *hmd and mer*		K00200, K00201, K00202, K00203, K11261, K00205, K11260, K00204, K00672, K01499, K00319, K13942, K00320
Acetate→methane	*acs*		K01895
Methanol→methane	*mtaA*, *mtaB*, *mtaC*		K14080, K04480, K14081
Methylamine/dimethylamine/trimethylamine→methane	*mtbA*, *mtmC*, *mtbC*, *mttC*, *mtmB*, *mtbB*, *mttB*		K14082, K16177, K16179, K14084, K16176, K16178, K14083
**Nitrogen Metabolism**			
Assimilatory nitrate reduction	*nasA*,*nasB*, *narB*, *NR*, *nirA*,*NIT-6*		K00372, K00360, K00367, K10534, K00366, K17877
Dissimilatory nitrate reduction	*nirB*, *nirD*, *nrfA*, *nrfH*, *nirk*, *nirS*, *norB*, *norC*, *nosZ*		K00362, K00363, K03385, K15876, K00368, K15864, K04561, K02305, K00376
Denitrification	*nirk*, *nirS*, *norB*, *norC*, *nosZ*		K00368, K15864, K04561, K02305, K00376
Nitrification	*hao*, *PmoA-amoA-C*		K10535, K10944, K10945, K10946
Nitrogen fixation	*nifD*,*nifK*, *nifH*, *anfG*, *vnfD*, *vnfk*, *vnfG*, *vnfH*		K02586, K02591, K02588,K00531,K22986, K22987, K22898, K22899
Anammox	*hdh*		K20935
**Sulfur Metabolism**			
Assimilatory sulfate reduction	*cysC*,*cysH*, *cysJ*, *cysI*,*Sir*		K00860, K00390, K00380, K00381,K00392
Dissimilatory sulfate reduction and oxidation	*aprA*, *aprB*,*dsrA*,*dsrB*		K00394, K00395,K11180, K11181
**Carbon fixation**			
3-hidroxypropionate bi-cycle	*accA*, *accB*, *accC*, *accD*,*mcr*,*mct*,*meh*,*smtA1*, *smtB*		K01962, K02160, K01961, K01963, K14468, K14469, K15052, K14470, K09709, K14471, K14472
Dicarboxylate-hidroxybutyrate cycle	*4hbl*		K14467
Reductive citrate cycle (Arnon-Buchanan cycle)	*pycA*,*pycB*, *pyc*, *frdA*, *frdB*, *frdC*, *frdD*,*frdE*, *aclA*, *aclB*, *ccsA*, *ccsB*, *ccl*		K01959, K01960, K01958, K18556, K18557, K18558, K18559, K18560, K15230, K15231, K15232, K15233, K15234
Calvin cycle	*prkB*, *rbcL*, *rbcS*, *GAPA*, K0110		K00855, K01601, K01602, K05298, K01100
Hidroxypropionate-hidroxybutylate	K15039, K15018, K15019, K15020, K14466		K15039, K15018, K15019, K15020, K14466
Reductive-CoA (Wood-lungdahl)	*cooS*, *fdhA*, *fdhB*, *metF*, *acsE*, *acsB*		K00198, K05299, K15022, K00297, K15023, K14138
**Photosynthesis**			
Photosystem I	*psaA*,*psaB*,*psaC*, *psaD*,*psaE*,*psaF*, *psaG*		K02689, K02690, K02691, K02692, K02693, K02694, K08905
Photosystem II	*psbA*,*psbB*,*psbC*, *psbD*,*psbE*,*psbF*, *psbL*		K02703, K02704, K02705, K02706, K02707, K02708, K02703
Allophycocyanin	*apcA*,*apcB*,*apcC*, *apcD*,*apcE*,*apcF*		K02092, K02093, K02094, K02095, K02096, K02097
Phycocyanin	*cpcA*,*cpcB*,*cpcC*, *cpcD*,*cpcE*,*cpcF*,*cpcG*, *pecA*,*pecB*,*pecC*, *pecE*,*pecF*		K02284, K02285, K02286, K02287, K02288, K02289, K02290, K02628, K02629, K02630, K02631, K02632
Phycoerythrin	*cpeA*, *cpeB*, *cpeC*,*cpeD*,*cpeE*,*cpeR*, *cpeS*,*cpeT*,*cpeU*,*cpeY*, *cpeZ*		K05376, K05377, K05378, K05379, K05380, K05381, K05382, K05383, K05384, K05385, K05386
LHC-antenna	*Lhca1*,*lhca2*,*lhca3*,*lhca4*,*lhca5*,*lhcb1*,*lhcb2*,*lhcb3*, *lhcb4*, *lhcb5*,*lhcb6*,*lhcb7*		K08907, K08908, K08909, K08910, K08911, K08912, K08913, K08914, K08915, K08916, K08917, K14172

Genes associated to each pathway and KO code are listed.

Potential pathways related to carbonate precipitation were searched within the metagenome. The pathways searched were: photosynthesis, ureolysis, denitrification, ammonification, sulfate reduction and methane oxidation [37]. This pathways were identified from the KO's code already obtained from the KEGG portal within the MG-RAST annotation. The microorganisms associated to each pathway were incorporated by the Ghost-Koala annotation.

### Nitrogen fixation and methanogenesis assays

To estimate the nitrogen fixation rates produced by *A*. *palmata* coral rubble, we included samples from the same points used for metagenomic analysis and blank controls to compare the production. Each of the replicates remained submerged inside incubation chambers (4.5 cm by 10 cm) in a tub (1.5 m diameter by 60 cm high) with constant water flow from the coastal lagoon maintaining *in situ* temperature and light conditions. The chambers had an aqueous section in the base (160 ml), where the samples were located, and a gaseous section in the surface (20 ml) that was sealed with rubber plugs and silicone. Once the chambers were sealed, nitrogenase activity was estimated with the acetylene reduction assay [38]. Each replicate was subjected to atmospheric saturation (20% of gas phase) with acetylene, which is reduced to ethylene by nitrogenase (C_2_H_2_ -> C_2_H_4_). Nitrogenase activity was monitored every 6 hours for 24 hours (5 total measurements counting initial measure). For measuring methanogenesis, same experiment as nitrogen fixation was performed but only air was added to evaluate methane accumulation.

Three milliliters of the gas phase were collected and injected into vacuum tubes (triplicates), for later analysis with gas chromatography [39]. The gas samples obtained from the acetylene reduction assays and methanogenesis experiment were analyzed in a gas chromatograph (Varían 3300), provided with a flame ionization detector, with which the amount of ethylene (C_2_H_4_) or methane (CH_4_) were determined. No methane was detected. Nitrogen fixation rates were calculated as a function of the proportion of the area under the curve between the sample and the standard (100 ppm), adjusted to minutes and the slope of the gas standard curve. Then the values ​​of the blanks were subtracted to correct planktonic rates of nitrogen fixation. The units obtained are in nanomoles of nitrogen per hour, which were normalized by organic carbon. All measurements were carried out in the Eukaryotic Functional Genomics Laboratory of the Genomics Sciences Center, UNAM.

### Extraction of pigments

Samples were macerated using mortar and pestle with liquid nitrogen and kept in the dark. To obtain phycobiliproteins, a volume (1:1) of 0.1 M potassium phosphate buffer was used, vortexed and incubated at 4ºC for at least 2 hours. The tubes were centrifuged for 10 minutes at 8,000 *rpm* the supernatant was removed and deposited in a new 50 ml tube covered with aluminum foil. One volume of 90% cold acetone was added and samples were incubated at 4°C overnight and centrifuged again for 10 minutes at 8,000 *rpm*. The calibration for the readings of the samples was elaborated from the potassium phosphate buffer and acetone respectively. The absorbances were read with a USB4000 mini-spectrophotometer (Ocean Optics, USA) and a SpectraSuite software. To perform the calculation of phycobiliprotein and chlorophyll concentrations, organic carbon were used to standardize the data samples. The equation of Kursar et al. [40] and Jeffrey & Humphrey [41] was followed for each sample respectively. The analysis focused on the absorption variation between 400 nm to 750 nm. All pigment determinations were carried out in the Photobiology Laboratory of the Academic Reefs Systems Unit (RSU), UNAM (Puerto Morelos, QRoo).

## Results

The Puerto Morelos reef is characterized on the basis of its physicochemical variables as a typical Caribbean location with average salinity (36.34 UPS) and circumneutral pH (8.1). Dissolved nutrients are similar to those reported in other studies for the region (Table 2).

**Table 2:** Physicochemical characterization of the surface water and coral rubble biofilm (average of 3 sub-samples).

	Surface Water	Coral rubble biofilm
Salinity (UPS)	36.35 ± 0.05	-
pH	8.11 ± 0.02	-
N-NH4 (μM)	1.23 ± 0.16	-
N- NO3 (μM)	0.9 ± 0.104	-
N-NO2 (μM)	0.06 ± 0.003	-
P-PO4 (μM) = SRP	0.21 ± 0.002	-
SiO2(μM)	2.86 ± 0.878	-
DIN(μM)	2.2 ± 0.104	-
TN(μM)	10.211 ± 5.2	-
TP(μM)	4.51 ± 1.61	-
DIN: SRP	10.47	-
TN:TP	2.26	-
N organic (mg/g)	-	0.063 ± 0.007
C organic(mg/g)	-	0.702 ± 0.024
C organic: N organic	-	11.14
TP (mg/g)	-	0.33 ± 0.07
TN (mg/g)	-	33.41 ± 0.40
TC (mg/g)	-	371.36 ± 1.28
TN:TP	-	10.124
TC:TN	-	11.11
TC:TP	-	1125.33

Coral rubble was initially characterized based on biogeochemical parameters to understand their organic matter and elemental proportions (Table 2). We now show that coral rubble is rich in C, N, organic matter and P. The quantification of pigments and chlorophylls indicates the presence of phycoerythrin, phycocyanin and allophycocyanin (Table 3). Phycoerythrin is in greater concentration in the coral rubble followed by phycocyanin and allophycocyanin. Chlorophyll *a* was the most abundant pigment, followed by *chlb* and *chlc*.

**Table 3:** Pigments and chlorophylls concentration in coral rubble biofilms.

Coral rubble	Phycoerythrin (μg/g)	Phycocyanin (μg/g)	Allophycocyanin (μg/g)	Equation for calculation
	585.31 (±77.26)	165.15 (±50.94)	198.34 (±70.51)	Kursar et al (1983)
biofilms	Chla (μg/g)	Chlb (μg/g)	Chlc (μg/g)	Equation for calculation
	208.13 (±55.87)	37.14 (±1.5)	24.94 (±7.36)	Jeffrey and Humphrey et al (1975)

### Metagenomic composition of coral rubble

Metagenomic analysis recovered initially 168,585,058 sequences, of which 146,635,889 remained after quality filtering, which have an average length of approximately 151 bp and an average GC-content of 50.6%. Of the total sequences, about 11% were classified as unknown, 13% did not pass the quality control and 76% were annotated. Of the annotated sequences 1,153,764 (~ 1%) were associated with ribosomal RNA genes; 47,791,606 (~ 37.3%) were predicted protein sequences with known functions and 79,190,216 (~ 61.80%) were protein sequences with unknown function.

The metagenomic sequence dataset was dominated by Bacteria with 87.37% (11,048,161 sequences), followed by Eukaryota with 8.12% (1,026,844 sequences), Archaea 3.39% (428,679) and Viruses with 0.73% (92345). Around 0.39% were unclassified sequences.

**Fig 2:**
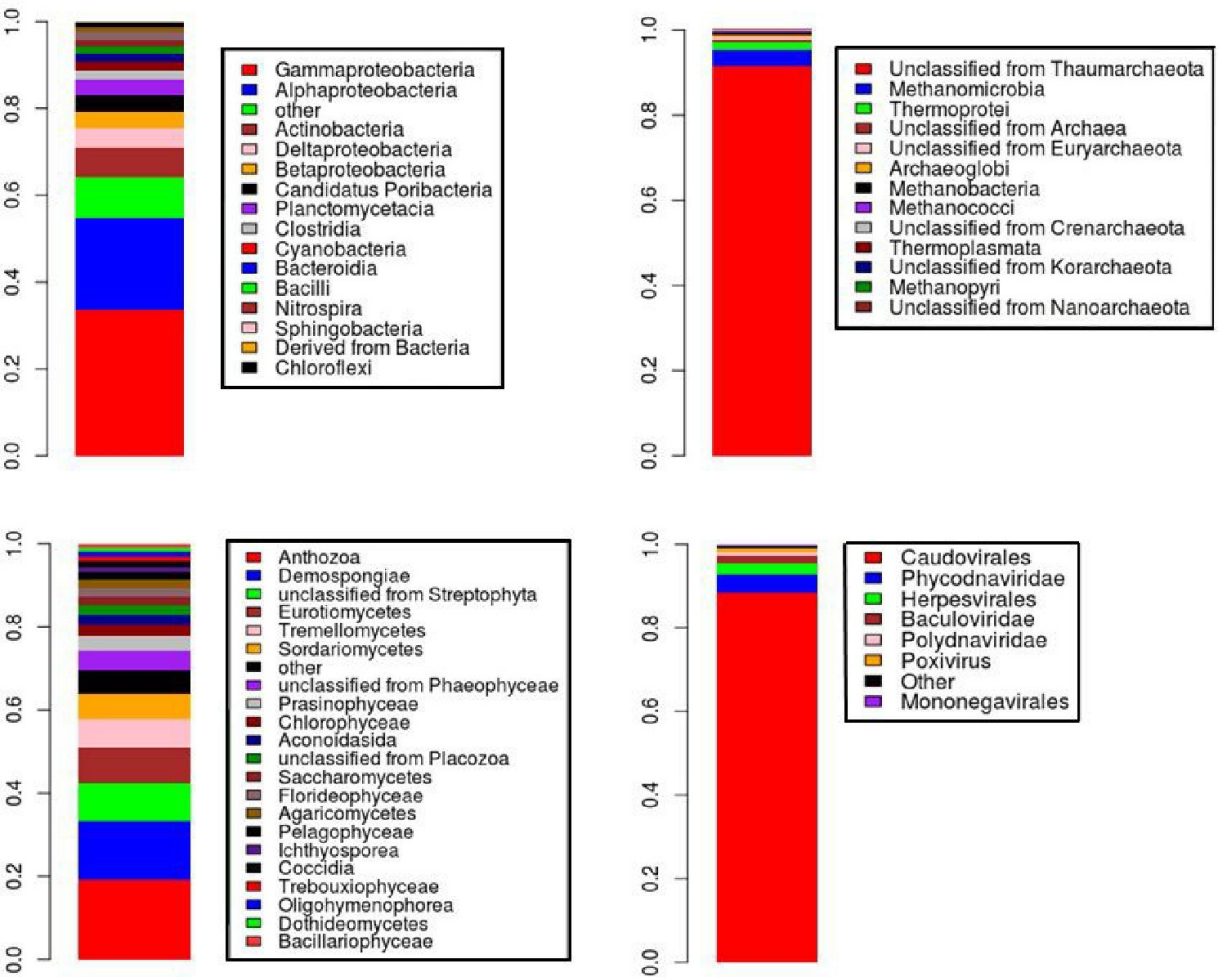
Phylogenetic composition of coral rubble for each domain. Bacteria 87.37% (A), Archaea 3.39% (B), Eukaryota 8.12% (C) and Virus 0.73% (D). A: Bacteria were dominated by Gammaproteobacteria (33.56%) and Alphaproteobacteria (21.36%). B: Archaea were dominated by unclassified Thaumarchaeota (91.54%). C: Eukaryotes were dominated by Anthozoa (19.00%) and Demospongiae (14.25%). D: Virus were dominated by Caudovirales (88.26%).

Within Bacteria, Gammaproteobacteria 33.56% was the most abundant class, followed by Alphaproteobacteria 21.36%, Actinobacteria 6.95%, Deltaproteobacteria 4.25%, Betaproteobacteria 3.98%, Candidatus Poribacteria 3.84%, Planctomycetacia 3.28%, Clostridia 2.32%, Cyanobacteria 1.93%, Bacteroidia 1.89%, Bacilli 1.65%, Nitrospira 1.59%, Sphingobacteria 1.55% and 1.37% unclassified bacteria. The most abundant genus in Bacteria were unclassified organisms from *Candidatus Poribacteria* 5.39%. Then *Chromohalobacter* 3.66%, *Halomonas* 2.84%, *Nitrospira* 2.00%, *Pseudomonas* 1.55%, *Prevotella* 1.46%, *Candidatus Solibacter* 1.19%, *Planctomyces* 1.17%, *Burkholderia* 1.15% and *Rhodothermus* 1.10% (Fig 2A).

The most abundant class in the Archaeal domain were unclassified Thaumarchaeota 91.54% (354,552), followed by Methanomicrobia 3.69% and Thermoprotei 1.75% (Fig 2B). The most abundant genus were *Nitrosopumilus*, *Cenarchaeum*, unclassified Thaumarchaeota, *Methanosarcina*, among others.

The most abundant Eukaryotes were Anthozoa 19.00% and Demospongiae 14.25%. Then followed by Streptophyta 9.07%, Eurotiomycetes 8.51%, Tremellomycetes 6.81%, Sordariomycetes 6.31%, Phaeophyceae 4.64%, Prasinophyceae 3.24%, Chlorophyceae 2.67%, Aconoidasida 2.45%, Placozoa 2.34%, Saccharomycetes 2.2%, Florideophyceae 1.99%, Agaricomycetes 1.92%, Pelagophyceae 1.75%, Ichthyosporea 1.41%, Coccidia 1.26%, Trebouxiophyceae 1.19%, Oligohymenophorea 1.16%, Dothideomycetes 0.97% and Bacillariophyceae 0.95%. The most abundant genus were *Filobasidiella*, *Monosiga*, *Ectocarpus* and *Penicillium* (Fig 2C).

The most abundant dsDNA bacteriophages in the coral-rubble metagenome were *Caudovirales* (88.26%) followed by Phycodnaviridae with 4.70% and Herpesvirales 2.63% (Fig 2D). The most abundant virus were *Microvirus*, Myoviridae, Siphoviridae and T4-like viruses.

### The potential metabolic role of the coral rubble from a metagenomic approach

**Fig 3:**
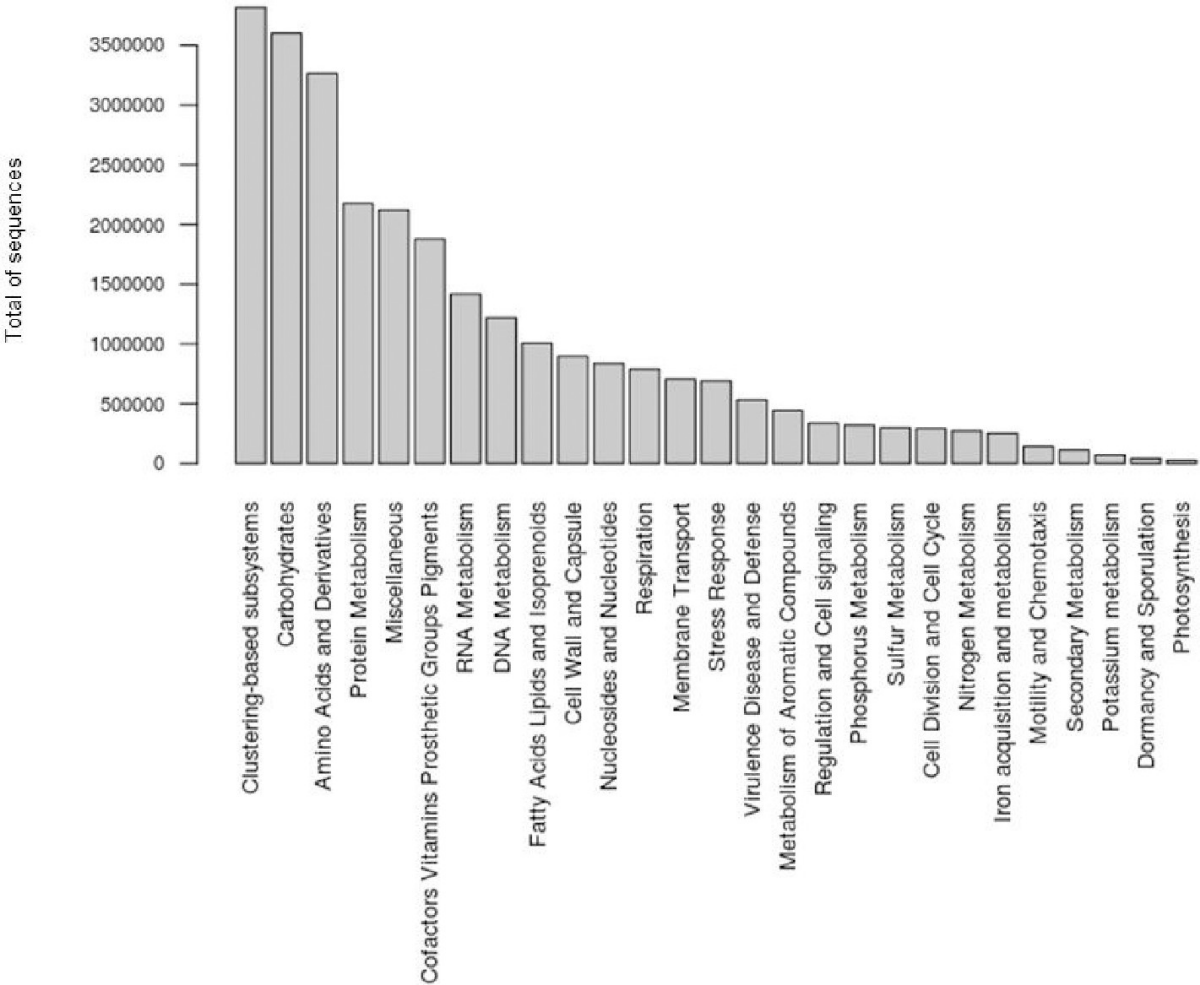
Metabolic potential of coral rubble. The metabolic potential of coral rubble is dominated by clustering-based subsystems (3,816,880 sequences) and carbohydrates (3,601,705 sequences). Amino acids and Derivatives, Protein metabolism and Miscellaneous (3,266,144; 2,177,104 and 2,122,139 sequences respectively) are also highly represented. Sequences coding for Potassium metabolism, Dormancy and Sporulation and Photosynthesis were represented by > 100,000 sequences.

The metabolic potential of the coral rubble was dominated by a clustering based subsystem (13.37%) and genes coding for core metabolic functions such as carbohydrate utilisation (12.6%), Amino Acids and Derivatives (11.44%), protein metabolism (7.62%), Miscellaneous (7.43%), Cofactors, Vitamins, Prosthetic Groups, Pigments (6.57%), RNA Metabolism (4.96%) and DNA metabolism (4.27%) (Fig 3).

**Fig 4:**
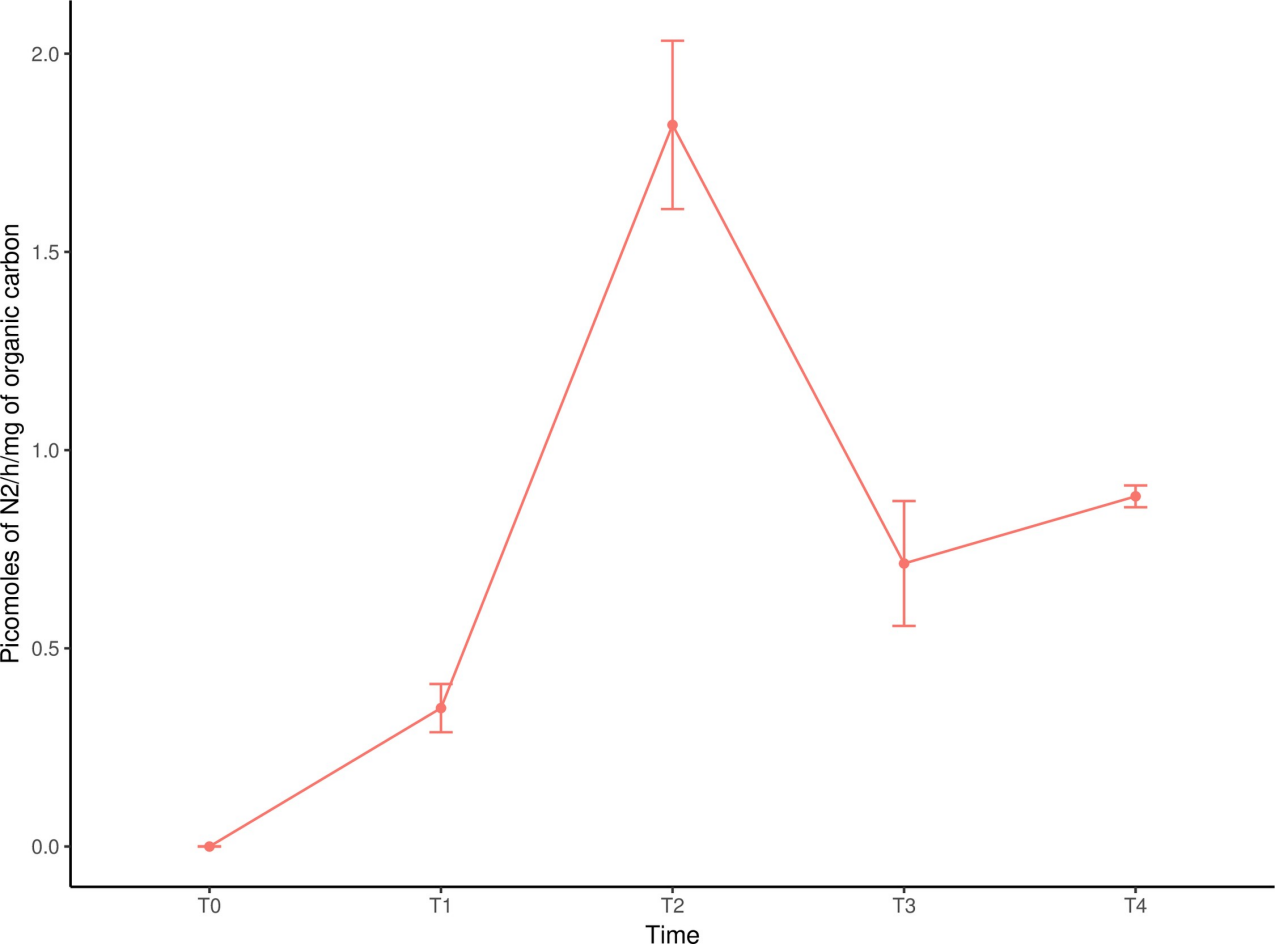
Nitrogen fixation quantified in coral rubble with the acetylene reduction assay over a 24h cycle. Assay started at 12:00h; T1 = 18:00h; T2 = 24:00h; T3 = 6:00h; T4 = 12:00h.

Coral rubble harbors a great diversity of bacteria which are associated to the elemental cycling of N, S, and C. Results suggest (S1 Table) that these communities are reducing nitrate and are involved in denitrification. Nitrogen fixation was quantified in coral rubble (Fig 4) with maximum activities between midnight and early morning. Although the same assays were carried for methanogenesis, no production was recorded. The identity of microorganisms associated to each genetic pathway are shown in S1 Table.

Photosynthetic potential was considered as the sum of the annotated sequences obtained from photosystems I and II in conjunction with the type of pigment that organisms possess (Phycobilisome: Allophycocyanin, Phycocyanin, Phycoerythrin vs LHC antenna—photosynthesis). The most abundant organisms were green algae, cyanobacteria, red algae and Stramenopiles. The most abundant genus were *Chondrus*, *Coccomyxa*, *Chlorella*, *Chlamydomonas*, *Ostreococcus*, *Aureococcus*, *Prochlorococcus*, among others (S1 Table).

The most abundant metabolisms related to carbonate precipitation were ammonification, sulfate reduction, denitrification, ureolysis, photosynthesis and methane oxidation (Table 4). Some microbial examples found per pathway were Cobetia and Nitrospira for ammonification. For sulfate reduction, Archaeoglobus and Candidatus Thioglobus were relevant. Nitrosopumilus and Gallionella within denitrification, Campylobacter in ureolysis and Synechococcus, Microcystis and Micromona in the photosynthetic pathway. Finally, Methylomicrobium was identified in the methane oxidation pathway.

**Table 4:** Metabolic pathways and organisms present in the coral rubble biofilms that have been associated with carbonate precipitation.

Metabolism	Number of hits in metagenome	Microbial groups	Identified groups	Example
Photosynthesis	4,027	photosynthetic organisms	Cyanobacteria & Algae	*Synechococcus*, *Microcystis* and *Micromonas*
Ureolysis	4,462	Ureolytic bacteria	Alphaproteobacteria & Epsilonproteobacteria	*Campylobacter*
Denitrification	5,828	Nitrate-reducing bacteria and archaea	Thaumarchaeota & Betaproteobacteria	*Nitrosopumilus* & *Gallionella*
Ammonification	53,660	Dissimilatory nitrate reduction bacteria	Gammaproteobacteria & Nitrospirae	*Cobetia* & *Nitrospira*
Sulfate reduction	38,457	Sulfate reduction bacteria and archaea	Proteobacteria, Firmicutes, Euryarchaeota	*Archaeoglobus* & *Candidatus Thioglobus*
Methane oxidation	478	Methanogens	Gammaproteobacteria	*Methylomicrobium*& *Methylococcus*

No carbonate precipitation measurements were done as part of this study. Hits from each pathway were obtained from MG-RAST annotation and organism from the Ghost-Koala annotation.

## Discussion

Current studies on coral rubble are scarce [17]. So far the coverage of dead coral rubble is a parameter seldom quantified in coral reef studies [7]. Nonetheless, these biofilm-covered structures are becoming conspicuous in certain reefs [7, 8, 12]. In the Mexican Caribbean, coral rubble has become a common sight of the reef landscape and so far, this is the first attempt to clarify their potential role in the environment. Previous reports have suggested that the microbial diversity of coral rubble is specific and different from healthy corals, surrounding water or sediment [17]. However, the potential role of coral rubble within the reef ecosystem had not been fully analyzed.

Reef biofilms are important as settlement cues for a variety of marine invertebrates, including corals [42, 43]. Nonetheless, coral rubble dominated- reefs such as La Bocana Grande in Mexico, do not sustain the settlement of coral larvae [22]. The potential role of coral rubble in reefs is poorly understood, this study represents an effort to deepen current knowledge on these fragile and highly vulnerable ecosystems. The data here reported consists of one metagenomic library, which was built from several subsamples representing the coral rubble diversity within La Bocana grande reef. We are aware that since samples were not separately indexed before pooling, the obtained results are less informative and may contain bias of the analysis. However, to our knowledge, this is the first metagenomic attempt to describe these communities and valuable information has been obtained. The functional role of microbes in coral reefs is becoming a new topic for investigation, since they play a fundamental role in the cycling of nutrients and energy on our planet [44]. The present metagenomic results point out the relevance of bacteria within the coral rubble, through their high abundance and involvement in several metabolic pathways.

Core metabolic functions including carbohydrate and protein metabolism dominated the coral rubble community. Carbohydrates and Protein metabolisms have an important role in energy storage within the coral rubble. Most microorganisms and algae can biosynthesize amino acids which have high representation in the metabolic results. Carbon, nitrogen and sulfur are essential and limiting nutrients for organisms in oceanic ecosystems [45]. Therefore, the ability for uptake of these nutrients may allow for marine organisms to survive in coral rubble ecosystems.

**Fig 5:**
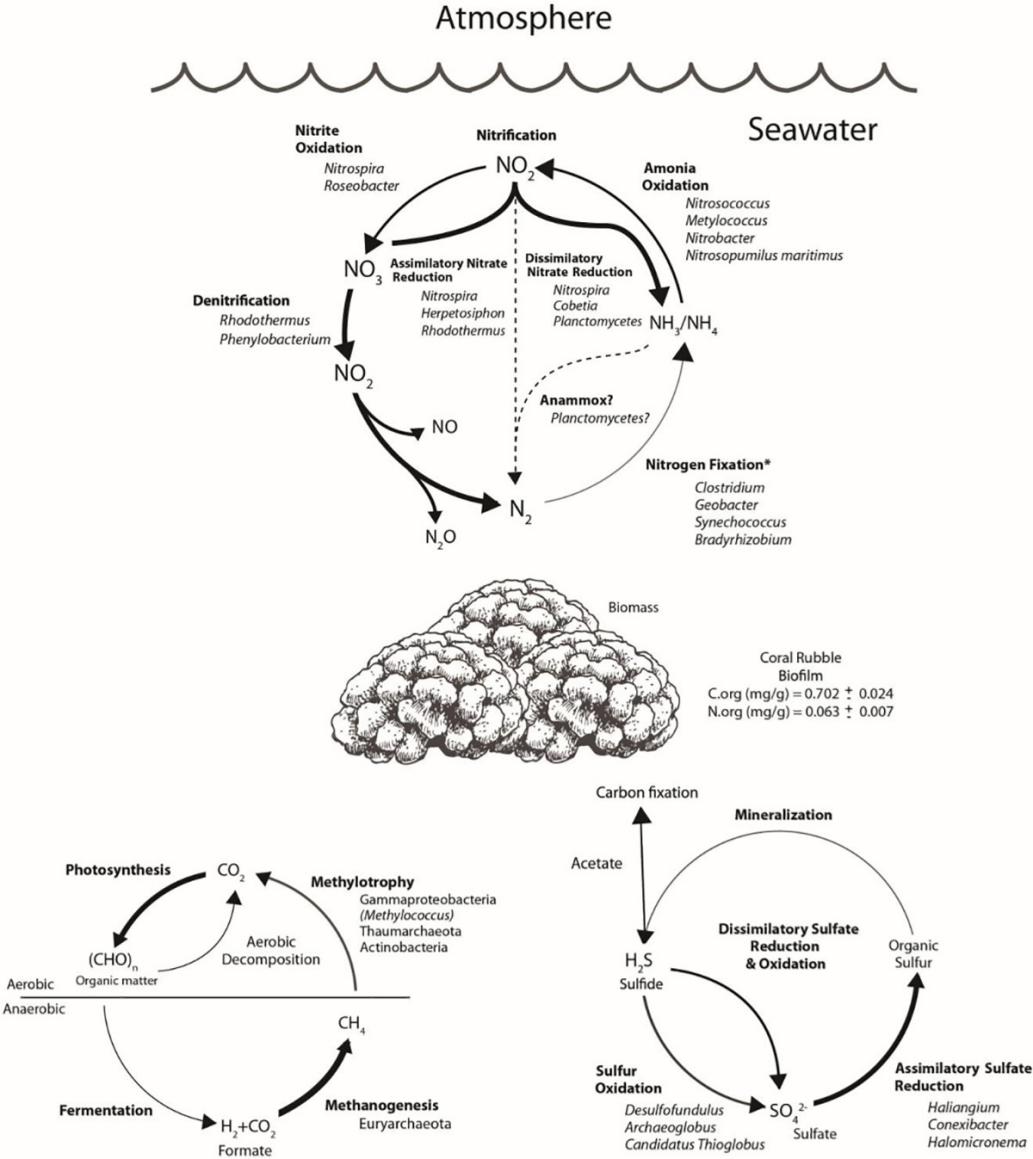
Model of the proposed functional role of bacteria in coral rubble assessed through metagenomics, indicating biogeochemical pathways associated to the nitrogen, sulfur and methane cycles. Arrows (bold) represent direction of pathway, sequence abundance, and (dotted) missing pathways. Phylogenetic identity and metabolic pathway genes were obtained from the MG-RAST and Ghost-Koala annotations.

Results suggest that coral rubble communities have an important role in nitrogen cycling through multiple pathways, where denitrification (5,928 hits), assimilatory and dissimilatory nitrate reduction (10,624 and 10,459 hits respectively) were more abundant. According to the metagenome total number of sequences, no apparent N limitation in the coral rubble is suggested (S1 Table and Fig 5). Hence, the incorporation of organic N and its remineralization stand out in this community. Archaea and Bacteria are likely to perform nitrification, in fact, according to Wuchter [46] *Nitrosopumilus maritimus* may dominate this process in seawater environments. According to the sequences obtained, nitrogen fixation is not a very abundant pathway. The *in situ* measurements of nitrogen fixation (Fig 4) revealed more nitrogenase activity between midnight and early morning, which coupled to the high numbers of nitrogenase-associated Alphaproteobacteria, suggests the role of heterotrophic diazotrophs in the coral rubble.

Recent studies have shown that coral reefs are likely to have an important role in biogeochemical cycling of sulfur [47–49]. Sulfur is found in seawater or sedimentary rocks, including calcium and magnesium carbonates [50]. Assimilatory sulfate reduction (30,871 hits) is the main pathway for the sulfur cycle in the assembled metagenome (S1 Table and Fig 5). This suggests that most sulfur is metabolized into organic compounds which are an essential component of proteins. The presence of sulfate-oxidizing bacteria, sulfate-reducing bacteria, sulfate reducing archaea (Euryarchaeota orders) and sulfur-dependent chemosynthesizer Crenarchaeota in the taxonomic metagenome annotation, implies a possible role for the coral rubble microbial community in the organic and inorganic sulfur cycling. Other organisms such as Actinobacteria, which was the third most abundant bacterial class, are related to the assimilation of sulphate, dissimilation of nitrate and methane metabolisms (data not shown). According to Anandan [51], Actinobacteria can also have an important role in the decomposition of organic matter and recycling of nutrients [51]. Thaumarchaeota, the most abundant phyla in Archaea, harbor ammonia-oxidizing chemolithotrophic organisms [52, 53] which were associated with nitrogen and carbon cycles. Anthozoa, Demospongiae and Streptophyta were the most abundant Eukaryotes which form part of the common reef diversity. Eurotiomycetes (Ascomycota) and Tremellomycetes (Basidiomycota) were fourth and fifth most abundant Eukaryota in the metagenomic assembly and have been reported to have potential roles in sulfur and nitrogen metabolism in coral reefs and marine environments [54–56].

Coral rubble is important in carbon cycling through methane transformations (S1 Table and Fig 5), where Euryarchaeota have a key role for methanogenesis. In fact, methanogenesis is only present in Archaea within the assembled metagenome. The rubble-associated methanotrophic archaea may play a role in methane transformation and C fixation. The presence of methanotrophs such as Euryarchaeota, Gammaproteobacteria and Thaumarchaeota suggests multiple methane transformation strategies within the community. Methane emission was not detectable *in situ*, although significantly more sequences hits were identified for methanogenesis than methane oxidation (S1 Table).

Microorganisms are fundamental in carbon cycling and they have important roles in different ecosystems. In coral rubble, all carbon fixation pathways were present in the assembled metagenome, indicating that these communities can fix CO_2_ and assimilate C under variable conditions. Alphaproteobacteria, Gammaproteobacteria, Cyanobacteria and Green algae are the main oxygenic photosynthesizers, although we can not discard the presence of anoxygenic phototrophs because they were part of the microbial diversity (S1 Table).

The coral rubble in La Bocana is a lithified biofilm formed by the action of coralline algae and microbes, that creates a secondary reef structure [17] where fragments of dead *A*. *palmata* are bound together through mineral precipitation. Metabolic pathways associated to carbonate minerals precipitation were identified in the coral rubble (Table 4). According to Riding [57] microorganisms can induce carbonate precipitation by altering solution chemistry or by serving as crystal nucleus [58]. In marine systems, photosynthetic microbes are responsible for triggering calcite precipitation [59]. Microbial carbonates produced by bacterial communities [16] and coralline algae, are important for the fixation of substrates [60] like coral rubble. Previous evidence has suggested that the surface of microbial biofilms can trap sediments and provide a medium for CaCO_3_ precipitation [17, 61]. The main carbonate mineralogies in shallow marine tropical waters are Mg-calcites, which are more abundant than aragonite in reefs [62]. Microbes can favor carbonate precipitation through different metabolic pathways. Within these metabolisms are photosynthesis, ureolysis, ammonification, denitrification, sulfate reduction, anaerobic sulfide oxidation, and methane oxidation [37]. These secondary reef structures could serve as substrate for corals or coralline algae to grow, although low coral coverage and renewal has been reported for La Bocana in Puerto Morelos [7, 22]. The rate and mechanisms of carbonate mineral precipitations in coral rubble merit future research.

## Conclusions

Coral rubble associated communities may play a significant role in coral reef environments through the remineralization of nitrogen, sulfur and carbon. Bacteria have a major role in coral-rubble, being the most abundant domain, where proteobacteria were the most abundant phylum and present in the majority of metabolic pathways. For future investigations, coral rubble from different reefs should be studied to understand if diversity is site-specific or if the metabolisms present in the coral rubble of La Bocana are a common feature in reefs that have high coral mortality. More comprehensive studies are required in order to discern the relative contributions of these rubble communities to the ecosystem. Understanding the diversity associated to coral rubble and the interactions in biogeochemical processes is essential to predict the functional changes occurring on coral reefs after coral death.

## Supporting information

S1 Table Metagenomic results for methane metabolism, nitrogen metabolism, sulfur metabolism, carbon fixation and photosynthesis. This table contains: pathways, searched genes (the number of hits per KO codes), total of hits per pathways and the presence of associated microorganisms per pathway. The KO's were quantified within the MG-RAST annotation. The Predominant organisms were obtained within the GHOST-KOALA annotation.
(DOCX)
