# Supplementary material for: Metagenome of Acropora palmata coral rubble: Potential metabolic pathways and diversity in the reef ecosystem
Source: PLoS One. 2019 Aug 8;14(8):e0220117. doi: 10.1371/journal.pone.0220117 (PMC6687439; doi:10.1371/journal.pone.0220117)
Supplement: S1 Table — This table contains: pathways, searched genes (the number of hits per KO codes), total of hits per pathways and the presence of associated microorganisms per pathway. The KO's were quantified within the MG-RAST annotation. The Predominant organisms were obtained within the GHOST-KOALA annotation. (DOCX) [file pone.0220117.s001.docx]

| **Energy metabolism** | Searched genes | KO code (#of hits in the metagenome) | Total hits per pathway | Predominant organisms (Phylum or Class: Genus) |
| --- | --- | --- | --- | --- |
| **Methane metabolism** |  |  |  |  |
| Methanotrophy | *pmoA-amoA-C mmo-X,Y,Z,B,C,D, mdh1,mdh2, MOX* | K10944(10), K10945(27), K10946(115), K16157(0), K16158(0), K16159(0), K16160(0), K16161(0), K16162(0), K14028(317), K14029(9), K17066(0) | 478 | Thaumarchaeota: Methylomicrobium, Methylococcus and Candidatus Nitrosomarinus |
| Methanogenesis | *mcrA, B, G, C, D* | K00399(3), K00401(0), K00402(0), K03421(0), K03422(0) |  | Euryarchaeota:Methanopyrus,Methanobacterium,Methanosphaera, Methanosphaera, Methanopyrus and Methanothermus |
| CO2→methane | *fwdA,B,C,D,E,F,G,H,ftr, mch, mtd, hmd* and *mer* | K00200(2347), K00201(506), K00202(187), K00203(8), K11261(36), K00205(0), K11260(0), K00204(0), K00672(1368), K01499(1163), K00319(2), K13942(0), K00320(3302) | 90,478 | Euryarchaeota: Methanotorris and Methanocaldococcus |
| acetate→methane | *acs* | K01895(66488) |  | Euryarchaeota: Methanococcoides and Methanoculleus |
| methanol→methane | *mtaA, mtaB, mtaC* | K14080(0), K04480(17), K14081(0) |  | Euryarchaeota: Methanomassiliicoccus and Methanosarcina |
| methylamine/dimethylamine/trimethylamine→methane | *mtbA, mtmC, mtbC, mttC,mtmB, mtbB, mttB* | K14082(2), K16177(0), K16179(0), K14084(210), K16176(0), K16178(0), K14083(14839) |  | Euryarchaeota: Methanomassiliicoccus, Candidatus Methanomethylophilus and Methanosarcina |
| **Nitrogen Metabolism** |  |  |  |  |
| Assimilatory nitrate reduction | *nasA,nasB, narB, NR, nirA, NIT-6* | K00372(8262), K00360(1000), K00367(267), K10534(10), K00366(1085), K17877(0) | 10,624 | Bacteroidetes, Cyanobacteria, Nitrospirae, Chloroflexi, Planctomycetes and Euryarchaeota:Nitrospira, Herpetosiphon, Rhodothermus, Phycisphaera |
| Dissimilatory nitrate reduction | *nirB, nirD, nrfA, nrfH* | K00362(9720), K00363(739), K03385(0), K15876(0) | 10,459 | Gammaproteobacteria, Actinobacteria, Planctomycetes Deltaproteobacteria, Nitrospirae, Betaproteobacteria,Alphaproteobacteria, Bacteriodetes and Firmicutes: Planctomyces, Nitrospira and Cobetia. |
| Denitrification | *nirk, nirS, norB, norC, nosZ* | K00368(2181), K15864(0), K04561(3170), K02305(477), K00376(1796) | 5,828 | Alphaproteobacteria,Gammaproteobacteria, Betaproteobacteria, Bacteroidetes, Deltaproteobacteria: Rhodothermus, Phenylobacterium, Gallionella, Methylomicrobium, Methylomonas. |
| Nitrification | *hao, PmoA- amoA-C* | K10535(497), K10944(10), K10945(27), K10946(115) | 649 | Gammaproteobacteria,Alphaproteobacteria,Thaumarchaeota, Actinobacteria, Betaproteobacteria, Nitrospirae, Firmicutes: Candidatus Nitrosomarinus, Nitrosomonas, Nitrobacter, Nitrospira, Nitrosopumilus |
| Nitrogen fixation | *nifD,nifK, nifH, anfG, vnfD, vnfk, vnfG, vnfH* | K02586(262), K02591(244), K02588(155), K00531(0), K22986(0), K22987(0), K22898(0), K22899(0) | 661 | Proteobacteria: Clostridium, Geobacter and Bradyrizobium |
| Anammox | *hdh* | K20935(0) | 0 | not found |
| **Sulfur Metabolism** |  |  |  |  |
| Assimilatory sulfate reduction | *cysC,cysH, cysJ, cysI,Sir* | K00860(6469), K00390(7154), K00380(821), K00381(15205), K00392(1222) | 30,871 | Gammaproteobacteria,Actinobacteria, Deltaproteobacteria, Thaumarchaeota, Cyanobacteria, Alphaproteobacteria, Bacteroidetes, Betaproteobacteria, Nitrospirae, Firmicutes, Ascomycetes:Conexibacter, Haliangium, Nitrospira, Nitrosopumilus, Halomicronema, Candidatus Nitrosomarinus, Cobetia, |
| Dissimilatory sulfate reduction and oxidation | *aprA, aprB, dsrA,dsrB* | K00394(4412), K00395(1044), K11180(1210), K11181(920) | 7,586 | Gammaproteobacteria,Firmicutes, Euryarchaeota,Alphaproteobacteria,Deltaproteobacteria,Thermodesulfobacteria:Archaeoglobus, Candidatus Thioglobus,Desulfofundulus,Syntrophobacter,Pseudodesulfovibrio,Thermodesulfobium, Thiohalobacter and Sulfuricaulis |
| **Carbon fixation** |  |  |  |  |
| 3-hidroxypropionate bi-cycle | *accA , accB, accC, accD,mcr, mct,meh, smtA1, smtB* | K01962(12939), K02160(3914), K01961(27913), K01963(11863), K14468(52), K14469(239), K15052(0), K14470(1270), K09709(921), K14471(266), K14472(714) | 60,091 | Chloroflexi |
| Dicarboxylate-hidroxybutyrate cycle | *4hbl* | K14467(12) | 12 | Crenarchaeota:Thermoproteus |
| Reductive citrate cycle (Arnon-Buchanan cycle) | *pycA,pycB, pyc, frdA, frdB, frdC, frdD,frdE, aclA, aclB, ccsA, ccsB, ccl* | K01959(6329), K01960(6939), K01958(14627), K18556(0), K18557(0), K18558(0), K18559(0), K18560(0), K15230(0), K15231(0), K15232(0), K15233(0), K15234(0) | 27,895 | Alphaproteobacteria, Nitrospirae, Deltaproteobacteria, Chlorobi: Nitrospira |
| Calvin cycle | *prkB, rbcL, rbcS, GAPA,* K01100 | K00855(2663), K01601(937), K01602(110), K05298(56), K01100(2) | 3,768 | Alphaproteobacteria, Gammaproteobacteria, Green algae, Cyanobacteria: Coccomyxa, Chondrus, Planctopirus and Chlorella |
| Hidroxypropionate-hidroxybutylate | K15039, K15018, K15019, K15020, K14466 | K15039(0), K15018(0), K15019(0), K15020(0), K14466(6) | 6 | Crenarchaeota:Metallosphaera, Sulfurisphaera and Sulfolobus |
| Reductive-CoA (Wood-lungdahl) | *cooS, fdhA, fdhB, metF, acsE, acsB* | K00198(244), K05299(0), K15022(0), K00297(8901), K15023(0), K14138(11) | 9,156 | Deltaproteobacteria, Euryarchaeota, Planctomycetes and Spirochaetes:Methanothrix |
| **Photosynthesis** |  |  |  |  |
| Photosystem I | *psaA,psaB, psaC,psaD, psaE,psaF, psaG* | K02689(616), K02690(688), K02691(49), K02692(55), K02693(18), K02694(60), K08905(0) | 1,486 | Green algae, Cyanobacteria, red algae and Stramenopiles:Chondrus, Coccomyxa, Chlorella, Chlamydomonas, Ostreococcus, Aureococcus, Prochlorococcus |
| Photosystem II | *psbA,psbB, psbC,psbD, psbE,psbF,psbF* | K02703(0), K02704(554), K02705(327), K02706(407), K02707(132), K02708(40)  K02703(0) | 1,460 | Green algae, Cyanobacteria, red algae and Stramenopiles:Chondrus, Coccomyxa, Chlorella, Chlamydomonas, Ostreococcus, Aureococcus, Prochlorococcus |
| Allophycocyanin | *apcA,apcB, apcC,apcD, apcE,apcF* | K02092(0), K02093(114), K02094(2), K02095(45), K02096(247), K02097(26) | 434 | Cyanobacteria: Thermosynechococcus, Synechococcus, Pleurocapsa, Gloeobacter, Synechococcus, Stanieria, Pseudanabaena and Microcystis |
| Phycocyanin | *cpcA,cpcB, cpcC,cpcD, cpcE,cpcF, cpcG, pecA,pecB, pecC, pecE,pecF* | K02284(48), K02285(73), K02286(59), K02287(0), K02288(37), K02289(5), K02290(71), K02628(0), K02629(0), K02630(0), K02631(4), K02632(0) | 297 | Cyanobacteria: Thermosynechococcus, Synechococcus, Pleurocapsa, Gloeobacter, Synechococcus, Stanieria, Pseudanabaena and Microcystis |
| Phycoerythrin | *cpeA, cpeB, cpeC,cpeD, cpeE,cpeR, cpeS,cpeT, cpeU,cpeY, cpeZ* | K05376(48), K05377(45), K05378(60), K05379(54), K05380(30), K05381(3), K05382(16), K05383(28), K05384(17), K05385(16), K05386(16) | 333 | Cyanobacteria: Thermosynechococcus, Synechococcus, Pleurocapsa, Gloeobacter, Synechococcus, Stanieria, Pseudanabaena and Microcystis |
| LHC-antenna | *Lhca1, lhca2,lhca3, lhca4,lhca5, lhcb1,lhcb2, lhcb3,lhcb4, lhcb5,lhcb6, lhcb7* | K08907(4), K08908(0), K08909(0), K08910(5), K08911(0), K08912(4), K08913(2), K08914(2), K08915(0), K08916(0), K08917(0), K14172(0) | 17 | Chlorophyta:Micromonas |
